# Identification and characterization of microRNAs expressed in the African malaria vector *Anopheles funestus* life stages using high throughput sequencing

**DOI:** 10.1186/s12936-016-1591-0

**Published:** 2016-11-08

**Authors:** Mushal Allam, Belinda L. Spillings, Hiba Abdalla, Darlington Mapiye, Lizette L. Koekemoer, Alan Christoffels

**Affiliations:** 1 SA Medical Research Council Bioinformatics Unit, South African National Bioinformatics Institute, University of the Western Cape, Robert Sobukwe Road, Cape Town, 7535 South Africa; 2 Sequencing Core Facility, National Institute for Communicable Diseases, National Health Laboratory Service, 1 Modderfontein Road, Johannesburg, 2131 South Africa; 3 Vector Control Reference Laboratory, Centre for Opportunistic, Tropical and Hospital Infections, National Institute for Communicable Diseases, National Health Laboratory Service, 1 Modderfontein Road, Johannesburg, 2131 South Africa; 4 Faculty of Health Sciences, Wits Research Institute for Malaria, University of the Witwatersrand, 1 Jan Smuts Ave, Johannesburg, 2000 South Africa; 5 Vector Biology & Control Unit, Blue Nile National Institute for Communicable Disease, Wad Medani, Sudan

**Keywords:** MicroRNA, Non-coding RNA, *Anopheles funestus*, Vector-borne diseases, Malaria

## Abstract

**Background:**

Over the past several years, thousands of microRNAs (miRNAs) have been identified in the genomes of various insects through cloning and sequencing or even by computational prediction. However, the number of miRNAs identified in anopheline species is low and little is known about their role. The mosquito *Anopheles funestus* is one of the dominant malaria vectors in Africa, which infects and kills millions of people every year. Therefore, small RNA molecules isolated from the four life stages (eggs, larvae, pupae and unfed adult females) of *An. funestus* were sequenced using next generation sequencing technology.

**Results:**

High throughput sequencing of four replicates in combination with computational analysis identified 107 mature miRNA sequences expressed in the *An. funestus* mosquito. These include 20 novel miRNAs without sequence identity in any organism and eight miRNAs not previously reported in the *Anopheles* genus but are known in non-anopheles mosquitoes. Finally, the changes in the expression of miRNAs during the mosquito development were determined and the analysis showed that many miRNAs have stage-specific expression, and are co-transcribed and co-regulated during development.

**Conclusions:**

This study presents the first direct experimental evidence of miRNAs in *An. funestus* and the first profiling study of miRNA associated with the maturation in this mosquito. Overall, the results indicate that miRNAs play important roles during the growth and development. Silencing such molecules in a specific life stage could decrease the vector population and therefore interrupt malaria transmission.

## Background

MicroRNAs (miRNAs) comprise a large family of endogenous, evolutionarily conserved, non-coding RNA that post-transcriptionally regulate gene expression in plants and animals [1, 2]. Many studies have indicated that miRNAs play a pivotal role in most critical biological events [3–17]. Therefore, identifying and characterization of miRNAs to understand their physiological and pathological roles have become popular research topics.

After the discovery of the first miRNA in the nematode, *Caenorhabditis elegans*, thousands of miRNAs have been identified [18–21]. The major approaches to identifying miRNAs include genetic screening, direct cloning, bioinformatic analysis, and deep sequencing [22, 23]. The majority of known miRNAs have been identified through traditional direct cloning, which is both time-consuming and labour-intensive. Bioinformatic analysis is limited because the majority of computational programs require genomic sequence as a template. Next generation sequencing (NGS) has provided an innovative tool to look into the genome with an unprecedented depth of coverage. It allows for a comprehensive coverage of miRNAs in many species [24–33]. As a consequence, this new approach has opened the door to functional genomic analyses of non-model species. It is widely used for profiling miRNAs in populations in various developmental stages, in either normal or diseased states [34–50].

Although there are over 30 species of *Anopheles* which transmit malaria in the world [51], miRNAs have so far only been experimentally identified in three malaria vectors, the African vector (*Anopheles gambiae*) and in two Asian vectors (*Anopheles stephensi* and *Anopheles anthropophagus*) [7, 52, 53]. Among the African vectors, *Anopheles funestus* is one of the most proficient malaria vectors, mainly because of its remarkable ability to populate a wide range of ecological settings across Africa [54–58]. Therefore, experimental identification of miRNAs controlling key genes required for mosquitoes to complete their life-cycle will not only help to better understand the vector biology, but it may uncover novel approaches to control this mosquito. In this context, the main objective of this study was to identify miRNAs expressed in the four main life stages (eggs, larvae, pupae and unfed adult females) of the mosquito *An. funestus* using high throughput sequencing technology and bioinformatics approaches.

## Methods

### Mosquito strain and rearing condition

The experimental work was performed using a colony of *An. funestus* (FUMOZ) that originates from southern Mozambique [59]. The mosquitoes were reared in the insectary of the Vector Control Reference Laborator at the National Institute for Communicable Diseases (NICD), Johannesburg, South Africa since 2000. The insectary is kept at 25 °C, 80% relative humidity with a 12-h day/night lighting regime and 45-min dusk/dawn cycles.

### RNA extraction and small RNA sequencing

Total RNA was isolated separately from the four different life cycle stages of *An. funestus* (eggs, larvae, pupae and unfed adult females) using TRIzol reagent (Invitrogen, USA) according to the manufacturer’s protocol. Four different biological replicates for each life stage for RNA extraction were prepared. In order to obtain a large and broad miRNA transcriptome data set, RNA was extracted from 5 egg patches, 100 fourth instar larvae, 100 pupae, and 100 unfed adult females for each single batch. The quality and quantity of resulting RNA was assessed using a spectrophotometer (Nanodrop Technologies), and quality assessment determined by using Agilent 2100 Bioanalyzer RNA Nano 6000 kit. No rRNA depletion was performed. The RNA extracts from the four life stages were sent to Macrogen Inc (South Korea) for small RNA sequencing. Sequencing libraries were generated from 1 μg each of the total RNA samples by ligation of adapter RNAs at 5′ and 3′ ends, followed by reverse transcription and PCR using Illumina TruSeq Small RNA Sample Preparation protocol (Illumina Inc., USA). The libraries were size-selected for sequencing of RNA fragments of 18–30 nucleotides. Sequencing was performed on an Illumina HiSeq 2000 platform to obtain single-end reads of 50 bases. Each batch sequenced independently.

### Sequencing data processing and analysis

#### Read quality check and filtering

Following sequencing, the quality of the four sequenced libraries were checked using FastQC [60]. Later, all sequencing reads with low quality tags and shorter than 18 nucleotides were removed using FASTX-Toolkit [61]. For all following analysis, all reads from each stage were combined into a single input.

#### Mapping the reads to the reference genome

All reads were mapped to the *An. funestus* strain FUMOZ genome [GenBank: KB668221]. The sequence reads were mapped to the genome using miRDeep2 mapper module [62].

#### Small non-coding RNAs detection

All reads were aligned to the RNA families database (Rfam) version 11 [63] using the BLASTn algorithm allowing for a 2 nucleotide mismatch and *e-value* lower than 0.01 in order to annotate the small non-coding RNAs present in the libraries.

#### Identification of miRNA sequences

For identification of miRNA sequences present in the four life stage datasets, the miRDeep2 package was employed [62]. The package scripts detect known or novel miRNAs from deep sequencing data. It looks for the pattern that the miRNA processing machinery leaves in the sequencing data. The most important pattern that miRDeep2 considers are clusters of reads that align along the reference genome that is compatible with the mature miRNA sequence, the loop sequence, and the star sequence structure of the miRNA precursor molecule. If such a pattern is found, miRDeep2 cuts out the potential miRNA precursor sequence from the reference genome and utilizes an RNA folding algorithm (randfold) from the Vienna package [64] to assess if the sequence can be folded into a hairpin structure. Furthermore, the prediction software searches for potential cleavage sites of *Drosha* and *Dicer*. The phylogenetic conservation and filtering of other known small non-coding RNA species can be optionally used to improve the predictions. All identified miRNAs are named according to their most similar miRNAs in the miRNA database (miRBase) release 21 (June 2014) [18–21] match.

#### Differential expression of the miRNAs

Differentially expressed miRNAs between two sequential life stages (egg-larva, larva-pupa, pupa-unfed adult female) were determined by log_2_ fold change >2 for the normalized reads +1. This analysis was computed using *gtools* package for the R programming language.

## Results

### Sequence quality of the four libraries

Small RNAs were sequenced in quadruplicate using Illumina sequencing from eggs, larvae, pupae and unfed adult females batches to identify *An. funestus* miRNAs expressed during development. More than 150 million raw reads were produced from each life stage (Table 1). The length distribution of the reads was between 18 and 30 nucleotides (data not shown). After filtering the impurities and reads of length smaller than 18 nucleotides, 75.92, 71.52, 80.85 and 75.39%, high-quality reads had Phred quality values (PQV) of 20 obtained from the eggs, larvae, pupae, and unfed adult females libraries, respectively (Table 1). The PQV has previously been reported to be an indicator of base call accuracy and therefore sequence quality [65].

**Table 1:** Summary of small RNA sequencing data analysis for the four life stage libraries of *An. funestus*

Stage	Eggs	Larvae	Pupae	Unfed adult females
Total raw reads (%)	151,229,067	165,299,940	165,782,104	154,104,200
High quality reads (%)	114,821,916 (75, 92)	118,232,206 (71, 52)	134,044,428 (80, 85)	116,182,404 (75, 39)
Mapped reads to the genome (%)	71,894,278 (62, 61)	75,552,909 (63, 90)	98,996,560 (73, 85)	69,037,524 (59, 42)

### Mapping reads from the four libraries

Mapping reads over the unmasked genome represents an unbiased option, allowing the detection of known and still undiscovered miRNAs. The total number of the reads mapped to *An. funestus* genome constitutes only 62.61, 63.90, 73.85 and 59.42% of the total high-quality reads from eggs, larvae, pupae, and unfed adult females libraries, respectively (Table 1).

**Fig. 1:**
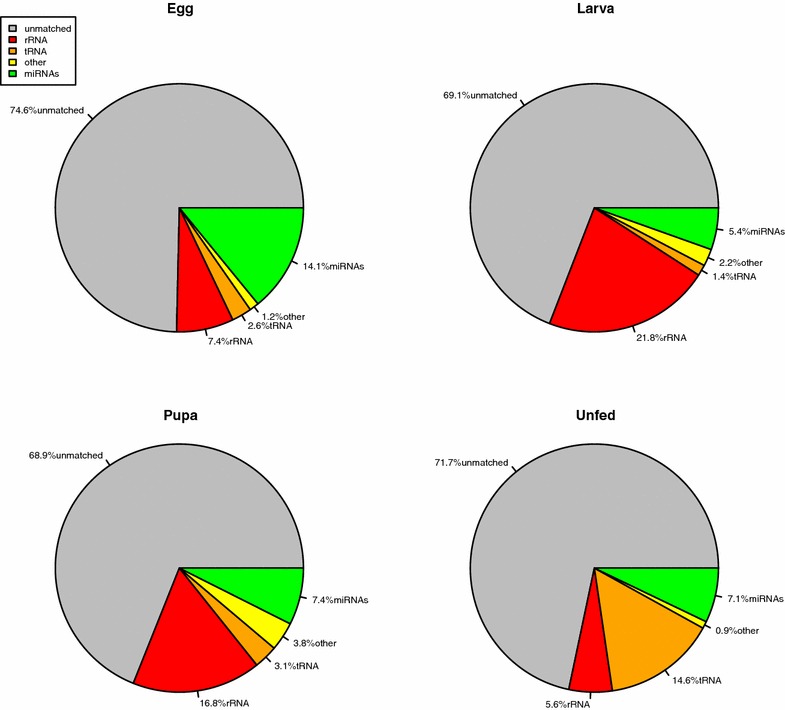
Small non-coding RNAs annotated from the four life stage libraries of *An. funestus*. From aligning the high quality reads to the RNA families database (Rfam) version 11. The total reads can be divided into five categories; miRNAs, tRNAs, rRNAs, others and unmatched. The others and unmatched referred to the other class of small non-coding RNAs and the reads not aligned to RNA families database , respectively

### Annotation of small non-coding RNAs in the four libraries

In order to annotate other small non-coding RNAs in the four libraries, all clean reads were aligned against Rfam version 11. As shown in Fig. 1, the most abundant class of small non-coding RNAs in the eggs library were miRNAs. However, rRNAs were most abundant in the larvae and pupae, and tRNAs in the unfed adult females library.

**Table 2:** Known miRNAs identified in the four life stages libraries of *An. funestus*

miRNA name	Sequences	Length	Location	Egg	Larva	Pupa	Unfed adult females
afu-bantam	UGAGAUCACUUUGAAAGCUGAU	22	KB669192.1_supercont1.62:255022..255087	$\surd$ $\surd$ $\surd$	$\surd$ $\surd$ $\surd$	$\surd$ $\times$ $\surd$	$\surd$ $\times$ $\surd$
afu-let-7	UGAGGUAGUUGGUUGUAUAGU	21	KB669047.1_supercont1.49:698595..698654	$\surd$ $\times$ $\surd$	$\surd$ $\surd$ $\surd$	$\surd$ $\times$ $\surd$	$\surd$ $\times$ $\surd$
afu-miR-1	UGGAAUGUAAAGAAGUAUGGAG	22	KB668556.1_supercont1.130:34984..35060	$\surd$ $\surd$ $\surd$	$\surd$ $\times$ $\surd$	$\surd$ $\surd$ $\surd$	$\surd$ $\times$ $\times$
afu-miR-10	ACCCUGUAGAUCCGAAUUUGUU	22	KB668870.1_supercont1.33:182453..182515	$\surd$ $\times$ $\surd$	$\surd$ $\times$ $\surd$	$\surd$ $\times$ $\surd$	$\surd$ $\times$ $\surd$
afu-miR-100	AACCCGUAGAUCCGAACUUGUG	22	KB669047.1_supercont1.49:703012..703075	$\surd$ $\surd$ $\surd$	$\surd$ $\surd$ $\surd$	$\surd$ $\surd$ $\surd$	$\surd$ $\surd$ $\surd$
afu-miR-1000	AUAUUGUCCUGUCACAGCAGUA	22	KB669169.1_supercont1.6:205456..205544	$\surd$ $\surd$ $\surd$	$\surd$ $\surd$ $\surd$	$\surd$ $\surd$ $\surd$	$\surd$ $\surd$ $\surd$
afu-miR-11	CAAGAACUUGGUACUGUGACCUGUG	25	KB669169.1_supercont1.6:410485..410557	$\surd$ $\surd$ $\surd$	$\surd$ $\times$ $\surd$	$\surd$ $\surd$ $\surd$	$\surd$ $\surd$ $\surd$
afu-miR-1175	AAGUGGAGUAGUGGUCUCAUCGCU	24	KB669358.1_supercont1.77:114656..114718	$\surd$ $\times$ $\surd$	$\surd$ $\times$ $\surd$	$\surd$ $\times$ $\surd$	$\surd$ $\times$ $\surd$
afu-miR-124	UAAGGCACGCGGUGAAUGCCA	21	KB668556.1_supercont1.130:267597..267658	$\surd$ $\times$ $\surd$	$\surd$ $\times$ $\surd$	$\surd$ $\times$ $\surd$	$\surd$ $\times$ $\surd$
afu-miR-125	UCCCUGAGACCCUAACUUGUGAC	23	KB669047.1_supercont1.49:697981..698042	$\surd$ $\times$ $\surd$	$\surd$ $\times$ $\surd$	$\surd$ $\surd$ $\surd$	$\surd$ $\times$ $\surd$
afu-miR-133	UUGGUCCCCUUCAACCAGCUGU	22	KB668872.1_supercont1.331:18310..18396	$\surd$ $\surd$ $\surd$	$\surd$ $\times$ $\surd$	$\surd$ $\times$ $\surd$	$\surd$ $\surd$ $\surd$
afu-miR-137	UAUUGCUUGAGAAUACACGUAG	22	KB669203.1_supercont1.63:461201..461262	$\surd$ $\times$ $\surd$	$\surd$ $\times$ $\surd$	$\surd$ $\times$ $\surd$	$\surd$ $\times$ $\surd$
afu-miR-13b	UAUCACAGCCAUUUUGACGA	20	KB668788.1_supercont1.256:85269..85330	$\surd$ $\times$ $\surd$	$\surd$ $\surd$ $\surd$	$\surd$ $\times$ $\surd$	$\surd$ $\times$ $\surd$
afu-miR-14	UCAGUCUUUUUCUCUCUCCUAU	22	KB668222.1_supercont1.10:1664927..1664991	$\surd$ $\surd$ $\surd$	$\surd$ $\times$ $\surd$	$\surd$ $\times$ $\surd$	$\surd$ $\times$ $\surd$
afu-miR-184	UGGACGGAGAACUGAUAAGGGC	22	KB668984.1_supercont1.432:34990..35049	$\surd$ $\times$ $\surd$	$\surd$ $\times$ $\surd$	$\surd$ $\times$ $\surd$	$\surd$ $\times$ $\surd$
afu-miR-1890	UGAAAUCUUUGAUUAGGUCUGG	22	KB668768.1_supercont1.238:330029..330096	$\surd$ $\surd$ $\surd$	$\surd$ $\surd$ $\surd$	$\surd$ $\surd$ $\surd$	$\surd$ $\surd$ $\surd$
afu-miR-1891	UGAGGAGUUAAUUUGCGUGUUU	22	KB668738.1_supercont1.210:313240..313298	$\surd$ $\times$ $\surd$	$\surd$ $\times$ $\surd$	$\surd$ $\times$ $\surd$	$\surd$ $\times$ $\surd$
afu-miR-190	AGAUAUGUUUGAUAUUCUUGGUUG	24	KB668683.1_supercont1.161:159767..159832	$\surd$ $\times$ $\surd$	$\surd$ $\times$ $\surd$	$\surd$ $\times$ $\surd$	$\surd$ $\times$ $\surd$
afu-miR-2-1/2	UAUCACAGCCAGCUUUGAUGAGC	23	KB668788.1_supercont1.256:84051..84114	$\surd$ $\surd$ $\surd$	$\surd$ $\surd$ $\surd$	$\surd$ $\times$ $\surd$	$\surd$ $\times$ $\surd$
afu-miR-210	CUUGUGCGUGUGACAACGGCUAU	23	KB668844.1_supercont1.306:43398..43456	$\surd$ $\times$ $\surd$	$\surd$ $\times$ $\surd$	$\surd$ $\times$ $\surd$	$\surd$ $\times$ $\surd$
afu-miR-219	AGAGUUGUGACUGGACAUCCGUG	23	KB668767.1_supercont1.237:131113..131179	$\surd$ $\surd$ $\surd$	$\surd$ $\surd$ $\surd$	$\surd$ $\times$ $\surd$	$\surd$ $\times$ $\surd$
afu-miR-263a	AAUGGCACUGGAAGAAUUCACGGG	24	KB668222.1_supercont1.10:692930..692993	$\surd$ $\times$ $\surd$	$\surd$ $\times$ $\surd$	$\surd$ $\times$ $\surd$	$\surd$ $\times$ $\surd$
afu-miR-263b	CUUGGCACUGGGAGAAUUCACAG	23	KB668812.1_supercont1.278:190692..190757	$\surd$ $\surd$ $\surd$	$\surd$ $\times$ $\surd$	$\surd$ $\times$ $\surd$	$\surd$ $\times$ $\surd$
afu-miR-275	UCAGGUACCUGAAGUAGCGCGCG	23	KB668400.1_supercont1.116:398864..398930	$\surd$ $\surd$ $\surd$	$\surd$ $\surd$ $\surd$	$\surd$ $\surd$ $\surd$	$\surd$ $\surd$ $\surd$
afu-miR-276	UAGGAACUUCAUACCGUGCUCU	22	KB668722.1_supercont1.197:198964..199032	$\surd$ $\surd$ $\surd$	$\surd$ $\surd$ $\surd$	$\surd$ $\times$ $\surd$	$\surd$ $\surd$ $\surd$
afu-miR-277	UAAAUGCACUAUCUGGUACGACA	23	KB669169.1_supercont1.6:1092927..1092992	$\surd$ $\surd$ $\surd$	$\surd$ $\surd$ $\surd$	$\surd$ $\surd$ $\surd$	$\surd$ $\surd$ $\surd$
afu-miR-278	ACGGACGAUAGUCUUCAACGACC	23	KB668223.1_supercont1.100:30655..30714	$\surd$ $\times$ $\surd$	$\surd$ $\surd$ $\surd$	$\surd$ $\times$ $\surd$	$\surd$ $\times$ $\surd$
afu-miR-279	UGACUAGAUCCACACUCAUUAA	22	KB668289.1_supercont1.106:353827..353890	$\surd$ $\times$ $\surd$	$\surd$ $\times$ $\surd$	$\surd$ $\times$ $\surd$	$\surd$ $\times$ $\surd$
afu-miR-281	AAGAGAGCUAUCCGUCGACAGU	22	KB669503.1_supercont1.90:359505..359565	$\surd$ $\times$ $\surd$	$\surd$ $\times$ $\surd$	$\surd$ $\times$ $\surd$	$\surd$ $\times$ $\surd$
afu-miR-282	UAGCCUCUUCUAGGCUUUGUCU	22	KB669503.1_supercont1.90:578663..578726	$\surd$ $\times$ $\surd$	$\surd$ $\times$ $\surd$	$\surd$ $\times$ $\times$	$\surd$ $\times$ $\times$
afu-miR-283	AAAUAUCAGCUGGUAAUUCUAGG	23	KB668836.1_supercont1.3:514580..514646	$\surd$ $\surd$ $\times$	$\surd$ $\surd$ $\surd$	$\surd$ $\surd$ $\times$	$\surd$ $\surd$ $\times$
afu-miR-286	UGACUAGACCGAACACUCGCGUCCU	25	KB668445.1_supercont1.120:344766..344839	$\surd$ $\surd$ $\surd$	$\surd$ $\times$ $\times$	$\surd$ $\times$ $\times$	$\surd$ $\times$ $\surd$
afu-miR-305	AUUGUACUUCAUCAGGUGCUCUGG	24	KB668400.1_supercont1.116:390085..390147	$\surd$ $\times$ $\surd$	$\surd$ $\times$ $\surd$	$\surd$ $\times$ $\surd$	$\surd$ $\times$ $\surd$
afu-miR-307	UCACAACCUCCUUGAGUGAGCGA	23	KB668223.1_supercont1.100:429991..430050	$\surd$ $\times$ $\surd$	$\surd$ $\times$ $\surd$	$\surd$ $\times$ $\surd$	$\surd$ $\times$ $\times$
afu-miR-308	CGCAGUAUAUUCUUGUGAACUUG	23	KB668933.1_supercont1.387:20015..20073	$\surd$ $\times$ $\surd$	$\times$ $\times$ $\times$	$\surd$ $\times$ $\surd$	$\surd$ $\times$ $\surd$
afu-miR-309	UCACUGGGCAAAGUUUGUCGCA	22	KB668445.1_supercont1.120:344139..344203	$\surd$ $\surd$ $\surd$	$\surd$ $\times$ $\times$	$\times$ $\times$ $\times$	$\times$ $\times$ $\times$
afu-miR-315	UUUUGAUUGUUGCUCAGAAAGCC	23	KB668747.1_supercont1.219:307337..307401	$\surd$ $\surd$ $\surd$	$\surd$ $\times$ $\surd$	$\surd$ $\times$ $\surd$	$\surd$ $\times$ $\surd$
afu-miR-317	UGAACACAUCUGGUGGUAUCUCAGU	25	KB669169.1_supercont1.6:1109058..1109126	$\surd$ $\surd$ $\surd$	$\surd$ $\surd$ $\surd$	$\surd$ $\surd$ $\surd$	$\surd$ $\surd$ $\surd$
afu-miR-34	UGGCAGUGUGGUUAGCUGGUUGU	23	KB669169.1_supercont1.6:1091079..1091145	$\surd$ $\times$ $\surd$	$\surd$ $\times$ $\surd$	$\surd$ $\times$ $\surd$	$\surd$ $\times$ $\surd$
afu-miR-375	UUUGUUCGUUUGGCUCGAGUUA	22	KB669281.1_supercont1.70:314839..314901	$\surd$ $\times$ $\times$	$\surd$ $\times$ $\surd$	$\surd$ $\times$ $\times$	$\surd$ $\times$ $\times$
afu-miR-7	UGGAAGACUAGUGAUUUUGUUGUU	24	KB668798.1_supercont1.265:312209..312271	$\surd$ $\surd$ $\surd$	$\surd$ $\times$ $\surd$	$\surd$ $\times$ $\surd$	$\surd$ $\times$ $\surd$
afu-miR-79	AUAAAGCUAGAUUACCAAAGCAU	23	KB668930.1_supercont1.384:9585..9649	$\surd$ $\surd$ $\surd$	$\surd$ $\surd$ $\surd$	$\surd$ $\times$ $\surd$	$\surd$ $\times$ $\surd$
afu-miR-8	UAAUACUGUCAGGUAAAGAUGUC	23	KB668666.1_supercont1.146:27512..27573	$\surd$ $\surd$ $\surd$	$\surd$ $\surd$ $\surd$	$\surd$ $\surd$ $\surd$	$\surd$ $\surd$ $\surd$
afu-miR-87	CCAGCCUGAAAUUUGCUAAACCU	23	KB669058.1_supercont1.5:469321..469384	$\surd$ $\surd$ $\surd$	$\surd$ $\surd$ $\surd$	$\surd$ $\times$ $\surd$	$\surd$ $\times$ $\surd$
afu-miR-927-5p	UUUAGAAUUCCUACGCUUUACC	22	KB668660.1_supercont1.140:64296..64359	$\surd$ $\surd$ $\surd$	$\surd$ $\times$ $\surd$	$\surd$ $\times$ $\surd$	$\surd$ $\surd$ $\surd$
afu-miR-929	AAAUUGACUCUAGUAGGGAGU	21	KB668836.1_supercont1.3:1318319..1318378	$\surd$ $\times$ $\surd$	$\surd$ $\times$ $\surd$	$\surd$ $\times$ $\surd$	$\surd$ $\times$ $\surd$
afu-miR-92a	UAUUGCACUUGUCCCGGCCUAU	22	KB668836.1_supercont1.3:1691007..1691065	$\surd$ $\times$ $\surd$	$\surd$ $\times$ $\surd$	$\surd$ $\times$ $\surd$	$\surd$ $\times$ $\surd$
afu-miR-92b	AAUUGCACUUGUCCCGGCCUGC	22	KB668836.1_supercont1.3:1704691..1704754	$\surd$ $\times$ $\surd$	$\surd$ $\times$ $\surd$	$\surd$ $\times$ $\surd$	$\surd$ $\times$ $\surd$
afu-miR-957	UGAAACCGUCCAAAACUGAGGC	22	KB669514.1_supercont1.91:212823..212889	$\surd$ $\times$ $\surd$	$\surd$ $\times$ $\surd$	$\surd$ $\times$ $\surd$	$\surd$ $\times$ $\surd$
afu-miR-970	UCAUAAGACACACGCGGCUAU	21	KB668797.1_supercont1.264:230410..230478	$\surd$ $\surd$ $\surd$	$\surd$ $\times$ $\surd$	$\surd$ $\times$ $\surd$	$\surd$ $\times$ $\surd$
afu-miR-981	UUCGUUGUCGACGAAACCUGCA	22	KB668389.1_supercont1.115:58097..58172	$\surd$ $\surd$ $\surd$	$\surd$ $\times$ $\times$	$\surd$ $\times$ $\times$	$\surd$ $\surd$ $\times$
afu-miR-988	CCCCUUGUUGCAAACCUCACGC	22	KB669391.1_supercont1.8:63805..63867	$\surd$ $\times$ $\surd$	$\surd$ $\times$ $\surd$	$\times$ $\times$ $\times$	$\times$ $\times$ $\times$
afu-miR-993	GAAGCUCGUUUCUAUAGAGGUAUC	24	KB668870.1_supercont1.33:220731..220822	$\surd$ $\surd$ $\surd$	$\surd$ $\times$ $\surd$	$\surd$ $\times$ $\surd$	$\surd$ $\times$ $\surd$
afu-miR-996	UGACUAGAUUACAUGCUCGUCU	22	KB668289.1_supercont1.106:354280..354344	$\surd$ $\surd$ $\surd$	$\surd$ $\times$ $\surd$	$\surd$ $\surd$ $\surd$	$\surd$ $\times$ $\surd$
afu-miR-9a	UCUUUGGUUAUCUAGCUGUAUGA	23	KB668816.1_supercont1.281:204094..204152	$\surd$ $\times$ $\surd$	$\surd$ $\surd$ $\surd$	$\surd$ $\times$ $\surd$	$\surd$ $\times$ $\surd$
afu-mir-9b	ACUUUGGUGAUUUAGCUGUAUGU	23	KB668930.1_supercont1.384:9150..9215	$\surd$ $\surd$ $\surd$	$\surd$ $\surd$ $\surd$	$\surd$ $\times$ $\surd$	$\surd$ $\times$ $\surd$
afu-miR-9c	UCUUUGGUAUUCUAGCUGUAGA	22	KB668930.1_supercont1.384:12370..12438	$\surd$ $\surd$ $\surd$	$\surd$ $\surd$ $\surd$	$\surd$ $\surd$ $\surd$	$\surd$ $\surd$ $\surd$
afu-miR-iab-4	ACGUAUACUGAAUGUAUCCUGA	22	KB668694.1_supercont1.171:357100..357157	$\times$ $\times$ $\times$	$\surd$ $\times$ $\times$	$\times$ $\times$ $\times$	$\surd$ $\times$ $\times$
afu-miR-12	UGAGUAUUACAUCAGGUACUGGU	23	Unknown	$\surd$ $\times$ $\times$	$\surd$ $\times$ $\times$	$\surd$ $\times$ $\times$	$\surd$ $\times$ $\times$
afu-miR-306	UCAGGUACUGGAUGACUCUCAG	22	Unknown	$\surd$ $\times$ $\times$	$\surd$ $\times$ $\times$	$\surd$ $\times$ $\times$	$\surd$ $\times$ $\times$
afu-mir-965	UAAGCGUAUAGCUUUUCCCAUU	22	Unknown	$\surd$ $\times$ $\times$	$\surd$ $\times$ $\times$	$\surd$ $\times$ $\times$	$\surd$ $\times$ $\times$
afu-miR-989	UGUGAUGUGACGUAGUGGUAC	21	Unknown	$\surd$ $\times$ $\times$	$\surd$ $\times$ $\times$	$\surd$ $\times$ $\times$	$\surd$ $\times$ $\times$
afu-miR-1174	UCAGAUCUACUUCAUACCCAUG	22	Unknown	$\surd$ $\times$ $\times$	$\surd$ $\times$ $\times$	$\surd$ $\times$ $\times$	$\surd$ $\times$ $\times$
afu-miR-1889	ACACAUUACAGAUUGGGAUUA	21	Unknown	$\surd$ $\times$ $\times$	$\surd$ $\times$ $\times$	$\surd$ $\times$ $\times$	$\surd$ $\times$ $\times$

The three columns for each life stage correspond to detection of the full precursor structure namely mature, loop and star sequence, respectively. $\surd$ $\surd$ $\surd$ indicates that miRNA full precursor structure was detected in the library where $\times$ $\times$ $\times$ indicates the the miRNA was not detected in the library

### Identification of miRNAs in the four life stages libraries of *An. funestus*

Of the 65 previously known *An. gambiae* mature miRNAs in miRBase release 21 (June 2014), 64 mature miRNAs were detected in the sequenced short reads of *An. funestus* (Table 2). The two *miR-276* precursors in *An. gambiae* generate two different mature miRNAs whereas in *An. funestus* they produce the same mature miRNA. These include all the 46 miRNA sequences computationally predicted in the *An. funestus* strain FUMOZ genome hosted by the invertebrate vectors of human pathogens database (VectorBase) [66]. The full precursor structure (mature, loop and star sequence) or parts of it were detected. Only star sequences were found for *miR-12*, *miR-306*, *miR-965*, *miR-989*, *miR-1174* and *miR-1889* and the precursor locations for these miRNAs were not determined. Reads that do not match any known *An. gambiae* miRNA sequences from miRBase and mapped to the *An. funestus* genome were screened for precursor or hairpin structures. The miRDeep2 algorithm was employed to investigate if non-annotated sequences mapping to the *An. funestus* genome demonstrated folding properties of precursors or hairpins. A total of 43 mature miRNA candidates expressed from 42 precursor candidates were identified (Table 3). Among these miRNAs, 15 miRNAs have been described in the *Anopheles* genus (*An. gambiae* and/or *An. anthropophagus*) but not registered in miRBase database, eight have not been previously reported in the *Anopheles* genus but are known in other mosquitoes such as *Aedes aegypti* and/or *Culex quinquefasciatus*, and the remaining 20 miRNAs have not been described before in any species. For all the mature miRNA candidates, the full or parts of the precursor were detected in the four life stage libraries except for *miR-2943* that was detected only in the eggs library. MiRNAs frequently exhibit sequence differences when compared to a reference mature sequence, generating multiple variations that are known as isoforms. New isoforms were identified for *miR-927* (*miR-927-3p*) and *miR-980* (*miR-980-5p*). The new isoforms of *miR-980* were expressed only in the eggs library. Our analysis uncovered a third stem-loop precursor for *miR-2*.

**Table 3:** Novel miRNAs identified in the four life stages libraries of *An. funestus*

miRNA name	Sequences	Length	Location	Egg	Larva	Pupa	Unfed adult females	Description	
afu-miR-2-3	UAUCACAGCCAGCUUUGAAGAGC	23	KB668788.1_supercont1.256:85435..85508	$\surd$ $\surd$ $\surd$	$\surd$ $\surd$ $\surd$	$\surd$ $\times$ $\surd$	$\surd$ $\times$ $\surd$	aae-miR-2a	Identified in *An. anthropophagus* and *An. gambiae* [53, 100]
afu-miR-252	CUAAGUACUAGUGCCGCAGGAG	22	KB669170.1_supercont1.60:199766..199821	$\surd$ $\times$ $\surd$	$\surd$ $\times$ $\surd$	$\surd$ $\times$ $\surd$	$\surd$ $\times$ $\surd$	dme-miR-252-5p	Identified in *An. anthropophagus* and *An. gambiae* [53, 100]
afu-miR-932	UCAAUUCCGUAGUGCAUUGCAGU	23	KB668896.1_supercont1.353:66093..66159	$\surd$ $\surd$ $\surd$	$\surd$ $\surd$ $\surd$	$\surd$ $\surd$ $\surd$	$\surd$ $\surd$ $\surd$	tca-mir-932	Identified in *An. anthropophagus* and *An. gambiae* [53, 100]
afu-miR-2796	GUAGGCCGGCGGAAACUACUUGC	23	KB669425.1_supercont1.83:106051..106111	$\surd$ $\times$ $\surd$	$\surd$ $\times$ $\surd$	$\surd$ $\times$ $\surd$	$\surd$ $\times$ $\surd$	bmo-miR-2796-3p	Identified in *An. anthropophagus* and *An. gambiae* [53, 100]
afu-miR-971	UUGGUGUUAUAUCUUACAGUGAG	23	KB669081.1_supercont1.52:713511..713586	$\surd$ $\surd$ $\surd$	$\surd$ $\surd$ $\surd$	$\surd$ $\surd$ $\times$	$\surd$ $\surd$ $\surd$	ame-miR-971	Identified in *An. gambiae* [53]
afu-miR-998	UAGCACCAUGAGAUUCAGCUC	21	KB669169.1_supercont1.6:410177..410245	$\surd$ $\surd$ $\surd$	$\surd$ $\surd$ $\surd$	$\surd$ $\surd$ $\surd$	$\times$ $\times$ $\times$	aae-miR-998	Identified in *An. gambiae* [53]
afu-mir-2942	UAUUCGAGACCUUCACGAGUUAA	23	KB668333.1_supercont1.11:1154487..1154559	$\surd$ $\surd$ $\surd$	$\surd$ $\surd$ $\surd$	$\surd$ $\times$ $\surd$	$\times$ $\times$ $\times$	aae-miR-2942	Identified in *An. gambiae* [53]
afu-miR-31	UAGCUAUUCAACUUCUUGUUUAU	23	KB668245.1_supercont1.102:383384..383442	$\surd$ $\times$ $\surd$	$\surd$ $\times$ $\surd$	$\surd$ $\times$ $\surd$	$\surd$ $\times$ $\surd$	cqu-miR-31-3p	Identified in *An. gambiae* [100]
afu-mir-33	GUGCAUUGUAGUUGCAUUGCA	21	KB668389.1_supercont1.115:38232..38297	$\surd$ $\surd$ $\surd$	$\surd$ $\surd$ $\surd$	$\surd$ $\times$ $\surd$	$\surd$ $\times$ $\surd$	aae-miR-33	Identified in *An. gambiae* [100]
afu-miR-285	UAGCACCAUUCGAAAUCAGUAC	22	KB668666.1_supercont1.146:348761..348825	$\surd$ $\times$ $\surd$	$\surd$ $\times$ $\surd$	$\surd$ $\times$ $\surd$	$\surd$ $\times$ $\surd$	aae-miR-285	Identified in *An. gambiae* [100]
afu-miR-980-5p	CGGUCGUUCACCAGGUCAUCUAGC	24	KB668730.1_supercont1.203:371780..371842	$\surd$ $\surd$ $\surd$	$\times$ $\times$ $\times$	$\times$ $\times$ $\times$	$\times$ $\times$ $\times$	aae-miR-980-5p	Identified in *An. gambiae* [100]
afu-miR-999	UGUUAACUGUAAGACUGUGUCU	22	KB668862.1_supercont1.322:158491..158552	$\surd$ $\times$ $\surd$	$\surd$ $\times$ $\times$	$\surd$ $\times$ $\times$	$\surd$ $\times$ $\times$	aae-miR-999	Identified in *An. gambiae* [100]
afu-miR-2940	GUCGACAGAGAGAUAGAUCACU	22	KB668668.1_supercont1.148:483999..484065	$\surd$ $\surd$ $\surd$	$\surd$ $\times$ $\surd$	$\surd$ $\surd$ $\surd$	$\surd$ $\times$ $\surd$	aae-miR-2940	Identified in *An. gambiae* [100]
afu-miR-2944a	GAAGGAACUUCUGCUGUGAUCU	22	KB668445.1_supercont1.120:344303..344359	$\surd$ $\times$ $\surd$	$\surd$ $\times$ $\surd$	$\surd$ $\times$ $\surd$	$\surd$ $\times$ $\surd$	aae-miR-2944a-5p	Identified in *An. gambiae* [100]
afu-miR-2944b	GAAGGAACUCCCGGUGUGAUAUA	23	KB668456.1_supercont1.121:76523..76595	$\surd$ $\surd$ $\surd$	$\surd$ $\times$ $\surd$	$\surd$ $\times$ $\surd$	$\surd$ $\times$ $\surd$	aae-miR-2944b-5p	Identified in *An. gambiae* [100]
afu-miR-71	UCUCACUACCUUGUCUUUCAUG	22	KB668788.1_supercont1.256:83429..83494	$\surd$ $\surd$ $\surd$	$\surd$ $\surd$ $\surd$	$\surd$ $\surd$ $\surd$	$\surd$ $\surd$ $\surd$	aae-miR-71-3p	Novel in *Anopheles spp.*
afu-miR-193	UACUGGCCUACUAAGUCCCAAC	22	KB668718.1_supercont1.193:152692..152757	$\surd$ $\surd$ $\surd$	$\surd$ $\surd$ $\surd$	$\surd$ $\surd$ $\surd$	$\times$ $\times$ $\times$	aae-miR-193	Novel in *Anopheles spp.*
afu-miR-316	UGUCUUUUCCUGCUUACUGCCG	22	KB668714.1_supercont1.19:760291..760355	$\surd$ $\times$ $\surd$	$\surd$ $\times$ $\surd$	$\surd$ $\times$ $\surd$	$\surd$ $\times$ $\surd$	aae-miR-316	Novel in *Anopheles spp.*
afu-miR-927-3p	UAAAGCGUAGGAAUUCUAAAAC	22	KB668660.1_supercont1.140:64294..64357	$\surd$ $\times$ $\surd$	$\surd$ $\times$ $\surd$	$\surd$ $\times$ $\times$	$\surd$ $\times$ $\surd$	dme-miR-927-5p	Novel in *Anopheles spp.*
afu-miR-980-3p	UAGCUGCCUAGUGAAGGGC	19	KB668730.1_supercont1.203:371784..371839	$\times$ $\times$ $\times$	$\surd$ $\times$ $\times$	$\surd$ $\times$ $\surd$	$\surd$ $\times$ $\times$	aae-miR-980-3p	Novel in *Anopheles spp.*
afu-miR-2765	UGGUAACUCCACCACCGUUGGC	22	KB668981.1_supercont1.43:346454..346513	$\surd$ $\times$ $\surd$	$\surd$ $\times$ $\surd$	$\surd$ $\times$ $\surd$	$\surd$ $\times$ $\times$	aae-miR-2765	Novel in *Anopheles spp.*
afu-miR-2941	UAGUACGGAUAAGUAACACACU	22	KB668992.1_supercont1.44:163365..163440	$\surd$ $\surd$ $\times$	$\surd$ $\times$ $\times$	$\surd$ $\times$ $\times$	$\times$ $\times$ $\times$	aae-miR-2941	Novel in *Anopheles spp.*
afu-miR-2943	UUAAGUAGGCACUUGCAGGCAA	22	KB668790.1_supercont1.258:358408..358469	$\surd$ $\surd$ $\surd$	$\times$ $\times$ $\times$	$\times$ $\times$ $\times$	$\times$ $\times$ $\times$	aae-miR-2943	Novel in *Anopheles spp.*
afu-miR-a	UGAGGUAAACCGCGCUUAAAGA	22	KB668367.1_supercont1.113:186839..186900	$\surd$ $\times$ $\surd$	$\surd$ $\times$ $\times$	$\surd$ $\times$ $\surd$	$\times$ $\times$ $\times$	-	Novel
afu-miR-b	AACGGACGGGUACCUUCGCACC	22	KB668478.1_supercont1.123:544003..544072	$\surd$ $\times$ $\times$	$\surd$ $\times$ $\times$	$\times$ $\times$ $\times$	$\times$ $\times$ $\times$	-	Novel
afu-miR-c	UCAACAUACAUUCUCGUUCUGU	22	KB668522.1_supercont1.127:174494..174560	$\times$ $\times$ $\times$	$\surd$ $\surd$ $\surd$	$\surd$ $\times$ $\surd$	$\times$ $\times$ $\times$	-	Novel
afu-miR-d	UAAUACAUUUGCUUACGGCAGAGU	24	KB668522.1_supercont1.127:49592..49655	$\surd$ $\times$ $\surd$	$\surd$ $\times$ $\surd$	$\times$ $\times$ $\times$	$\times$ $\times$ $\times$	-	Novel
afu-miR-e	UCUGCCGUAGGAAUGUAUUUACC	23	KB668522.1_supercont1.127:51191..51253	$\surd$ $\times$ $\surd$	$\surd$ $\times$ $\surd$	$\surd$ $\times$ $\surd$	$\surd$ $\times$ $\surd$	-	Novel
afu-miR-f	UGCUGUAGGAAGUGAUUUACCU	22	KB668522.1_supercont1.127:51545..51607	$\surd$ $\times$ $\surd$	$\surd$ $\times$ $\surd$	$\surd$ $\times$ $\surd$	$\surd$ $\times$ $\surd$	-	Novel
afu-miR-g	AGUCAUCGUCGUCACUCGAUCGA	23	KB668728.1_supercont1.201:241585..241642	$\surd$ $\times$ $\surd$	$\times$ $\times$ $\times$	$\surd$ $\times$ $\times$	$\surd$ $\times$ $\surd$	-	Novel
afu-miR-h	CCAUCUGACGGACACCACCGGA	22	KB668739.1_supercont1.211:143895..143961	$\surd$ $\times$ $\surd$	$\surd$ $\times$ $\surd$	$\times$ $\times$ $\times$	$\times$ $\times$ $\times$	-	Novel
afu-miR-i	AUGGCAGUCAACGUAUACCCAUU	23	KB668781.1_supercont1.25:515317..515377	$\surd$ $\times$ $\surd$	$\surd$ $\times$ $\surd$	$\surd$ $\times$ $\surd$	$\surd$ $\times$ $\surd$	-	Novel
afu-miR-j	UUCCGAGUGGACAAGUGGAACCU	23	KB668806.1_supercont1.272:68360..68416	$\surd$ $\times$ $\times$	$\surd$ $\times$ $\times$	$\surd$ $\times$ $\times$	$\times$ $\times$ $\times$	-	Novel
afu-miR-k	UACCUGAGUUCGUUUAAACUGA	22	KB668821.1_supercont1.286:244740..244793	$\surd$ $\times$ $\times$	$\surd$ $\times$ $\times$	$\surd$ $\times$ $\times$	$\surd$ $\times$ $\times$	-	Novel
afu-miR-l	UCUAUCAUUUGAGUACCAUGA	21	KB668837.1_supercont1.30:192923..192989	$\surd$ $\times$ $\surd$	$\surd$ $\times$ $\surd$	$\surd$ $\times$ $\surd$	$\surd$ $\times$ $\surd$	-	Novel
afu-miR-m	CUAACCUGUAAGGAGCUUUGGCGGC	25	KB668906.1_supercont1.362:184842..184933	$\times$ $\times$ $\times$	$\surd$ $\times$ $\surd$	$\surd$ $\times$ $\surd$	$\surd$ $\surd$ $\surd$	-	Novel
afu-miR-n	AUGGCACAAGCAACAUCAGCUG	22	KB668909.1_supercont1.365:72126..72172	$\times$ $\times$ $\times$	$\times$ $\times$ $\times$	$\surd$ $\times$ $\times$	$\surd$ $\times$ $\times$	-	Novel
afu-miR-o	UCUUGAGCGUAUUUGGCACUGCU	23	KB668992.1_supercont1.44:163580..163641	$\surd$ $\times$ $\surd$	$\surd$ $\times$ $\surd$	$\times$ $\times$ $\times$	$\surd$ $\times$ $\times$	-	Novel
afu-miR-p	UAGCUCAACAAACAUCUGGAGGA	23	KB669059.1_supercont1.50:521609..521669	$\surd$ $\times$ $\surd$	$\surd$ $\times$ $\surd$	$\surd$ $\times$ $\surd$	$\surd$ $\times$ $\surd$	-	Novel
afu-miR-q	UACAACCGGACGGUACACUGCAUAG	25	KB669214.1_supercont1.64:671164..671237	$\surd$ $\times$ $\surd$	$\surd$ $\surd$ $\surd$	$\surd$ $\times$ $\times$	$\times$ $\times$ $\times$	-	Novel
afu-miR-r	AACGAAGUUGAUCCGCUGAAGCU	23	KB669480.1_supercont1.88:541508..541571	$\surd$ $\times$ $\surd$	$\surd$ $\times$ $\surd$	$\surd$ $\times$ $\times$	$\times$ $\times$ $\times$	-	Novel
afu-miR-s	ACUUGAAACGUAGUCCGGGAACCC	24	KB669502.1_supercont1.9:563831..563898	$\surd$ $\times$ $\surd$	$\surd$ $\times$ $\times$	$\times$ $\times$ $\times$	$\times$ $\times$ $\times$	-	Novel
afu-miR-t	AUUAGAAUGUGGAAUCUGUUUUU	23	KB668820.1_supercont1.285:93655..93723	$\surd$ $\surd$ $\surd$	$\surd$ $\times$ $\surd$	$\surd$ $\times$ $\surd$	$\surd$ $\times$ $\surd$	-	Novel

The three columns for each life stage correspond to detection of the full precursor structure namely mature, loop and star sequence, respectively. $\surd$ $\surd$ $\surd$ indicates that miRNA full precursor structure was detected in the library where $\times$ $\times$ $\times$ indicates the the miRNA was not detected in the library

**Fig. 2:**
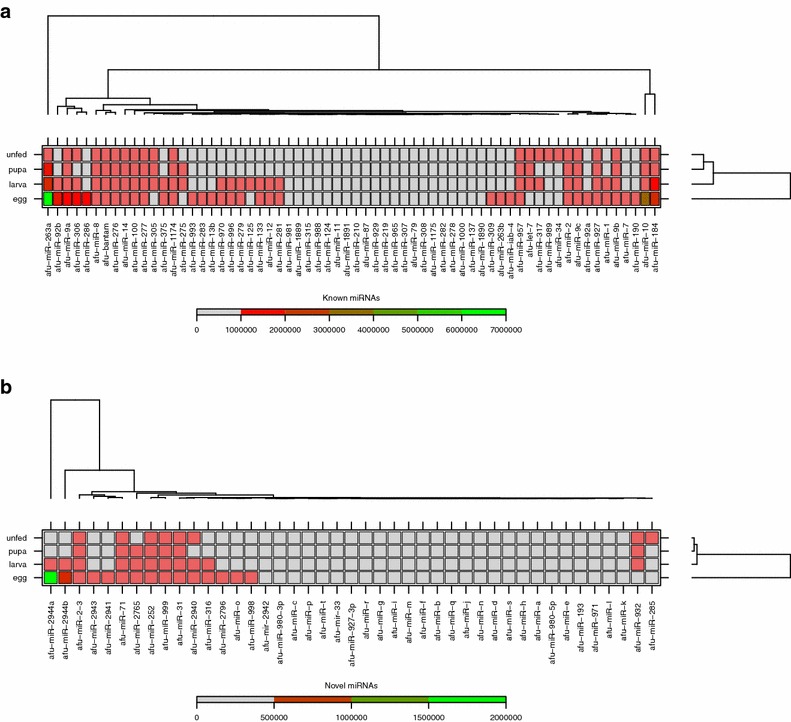
Heatmaps clustering of miRNAs expressed in the four life stage libraries of *An. funestus*. The clustering was performed on all known (**a**) and novel (**b**) miRNAs based on a raw read copy number (sequencing frequency) and the four life stage samples. Each* row* represents a stage and each column represents one miRNA. The miRNA clustering is shown on* top*. The* colour scale* (shown on the* left*) illustrates the number of the reads of a miRNA across the life stages

**Fig. 3:**
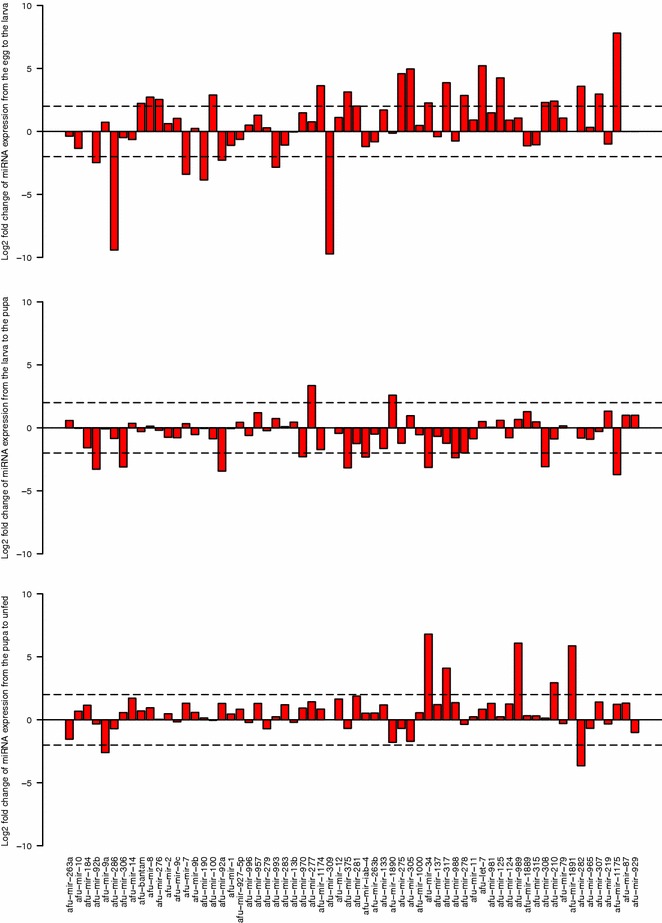
Fold-change of the known miRNAs during the development of *An. funestus*. The fold-changes were calculated for each miRNA (x-axis) using normalized reads +1 (y-axis,* bars*)

**Fig. 4:**
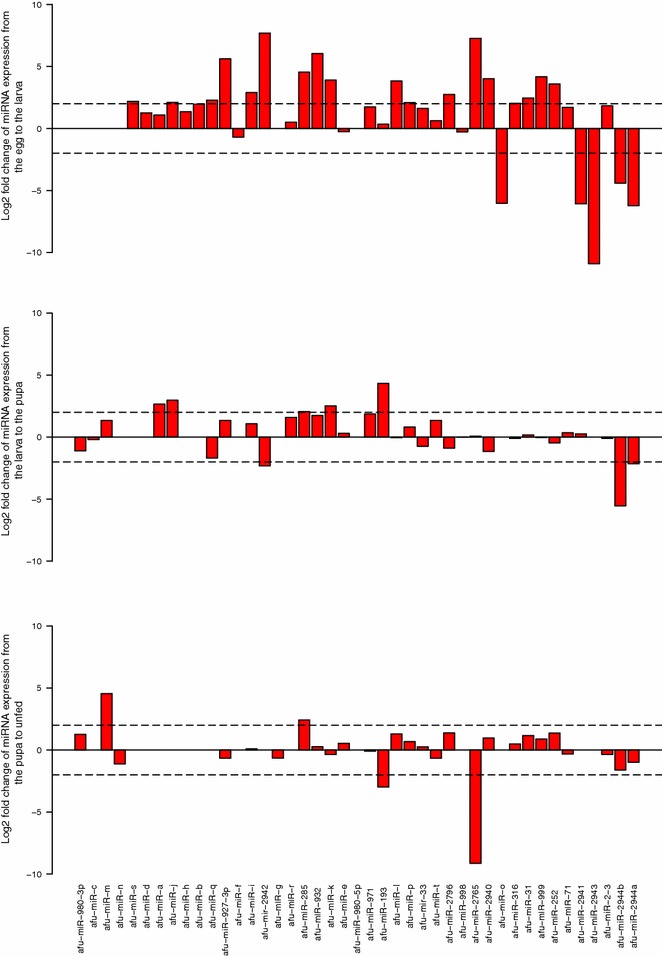
Fold-change of the novel miRNAs during the development of *An. funestus*. The fold-changes were calculated for each miRNA (x-axis) using normalized reads +1 (y-axis,* bars*)

### Expression profiling of miRNAs during *An. funestus* development

To obtain insight into possible stage dependent roles of miRNAs in *An. funestus*, the expression patterns of miRNAs in different life stages were examined based on the number of reads obtained. The heatmaps (Fig. 2) summarize the expression of the miRNAs identified in the four life stage libraries. The majority of miRNAs were sequenced between 1–6^6^ times. The expression profiles of miRNAs varied from highly specific to ubiquitous during the four stages. Among the known miRNAs, *miR-263a* was the most frequently expressed miRNA in the eggs, the larvae, and the pupae libraries (>1 million reads), whereas *miR-8* was the dominant miRNA in the unfed adult females library (>300 thousand reads). Nevertheless, *miR-87* had the lowest expression level in the egg and the pupa, similarly *miR-189* in the larva, and *miR-309* and *miR-929* in the unfed adult females library. The highly expressed miRNA amongst the new miRNAs in the eggs library was *miR-2944a* (>1.5 million reads), *miR-2765* (>20 thousand reads) in the larvae and pupae libraries and *miR-999* (>30 thousand reads) in the unfed adult females library. To analyse the changes in the expression of miRNAs during the mosquito life stages, all read counts within a dataset were normalized, then log2 (fold-changes) were calculated between each two stages. The results of the comparisons (egg-larva, larva-pupa and pupa-unfed adult female) of the expression level of all the miRNAs in the four libraries are shown in Figs. 3 and 4. Between the egg to the larva, 29 miRNAs were up-regulated and 12 other miRNAs were down-regulated. Six miRNAs were up-regulated and another 13 down-regulated between the larva and pupa stage. However, seven miRNAs were up-regulated and four down-regulated between the pupa and the unfed adult female.

## Discussion

Although thousands of small RNAs have been identified [18–21], the challenge remains to fully identify all small RNAs, especially very low abundance miRNAs and to determine their individual functions. The majority of known miRNAs have been identified through the traditional cloning method [67]. The Illumina sequencing approach is one of high throughput technologies by which miRNAs can be detected in any organism without prior sequence or secondary structure information sequence or secondary structure information. Here, 107 miRNA sequences expressed in the four life stages of the African malaria vector *An. funestus* were identify and characterize using the deep sequencing approach. This method is more powerful than other conventional technologies previously used in mosquitoes [7, 11, 52], as it is able to identify new miRNAs that are beyond the capabilities of older traditional methods.

In this study, approximately 100 million high-quality reads from each life stage by deep sequencing were obtained. The size distribution of sequenced reads showed peaks between 20–30 nucleotides compare to the average miRNA length of 22 nucleotides in animals [1]. Using similar deep sequencing techniques, other studies on insects show small RNA sequence size distributions with a peak at 22 nucleotides [11, 34, 68, 69]. All the high-quality reads were mapped on to the *An. funestus* genome with low percentage (average of 65%) identity. Such a mapping bias has been reported in several studies [21, 70–72]. This approach has the weakness that it might favour alignment ambiguities due to the limited alignment specificity given by the small length of mature miRNAs (18–25 nucleotides) detected by the short reads, and to the size and high complexity of an unmasked reference genome [70].

As expected, most of the known miRNAs identified in *An. funestus* were highly conserved across diverse animal species, suggesting that the ancient regulatory pathways mediated by evolutionary conserved miRNAs are present in mosquitoes. The full stem-loop precursor structure was detected in all four life stage libraries for *miR-8*, *miR-9c*, *miR-100*, *miR-275*, *miR-277*, *miR-317*, *miR-1000* and *miR-1890*. In the majority of cases, mature miRNAs are more abundant than loop and star sequences. Additionally, *miR-309* and *miR-988* were not detected in the pupae and unfed adult females libraries, *miR-iab-4* was not found in eggs and pupae libraries and *miR-308* was not identified from the larvae library. These results indicate that the expression of some miRNAs are probably stage specific. It can be speculated that a miRNA may be involved in regulation of function and dysfunction, differentiation, growth and development of a specific stage [73].

The identification of novel miRNAs is an eminent and challenging problem for the understanding of post-transcriptional gene regulation. The characteristic hairpin structure of miRNA precursors can be used to predict novel miRNAs. With this feature and using miRDeep2, novel miRNAs were predicted by exploring the secondary structure, the *Dicer* cleavage site, and the minimum free energy of the unannotated small RNA tags that could be mapped to the *An. funestus* genome. Based on the analysis, 43 mature sequences were identified as novel miRNA candidates because they were not captured in any RNA database. The full precursor structure for some of these novel miRNAs was identified. Detection of the miRNA star is a strong clue, albeit not infallible, for the formation of precursor hairpin structures. This adds weight to the authenticity of the predicted candidates [74, 75]. However, the evolution and function of the star miRNAs remains unclear. Two studies proposed that these star miRNAs might differ from their sense partners by acting on different mRNA targets [76, 77].

The miRBase databases release 21 (June 2014) was searched for homologs to determine whether these novel miRNAs are conserved among other animal species. Some candidates are conserved in other insect species but not in the *Anopheles* genus, suggesting that these are insect-specific miRNAs. Among the newly identified *Anopheles* miRNAs, four mosquito specific miRNAs (*miR-2940*, *miR-2941*, *miR-2942* and *miR-2943*) were detected [68]. This is the first description of *miR-2941* and *miR-2943* in the anopheline species using the high-throughput sequencing technology.

The NGS technology was sensitive enough to detect a new variant for *miR-927* and *miR-980*, miRNAs found only in insects. These results are congruent with the existence of multiple variants for both miRNAs in other insects such as *Drosophila* [18]. A third stem-loop precursor for *miR-2* was also detected which produces different mature *miR-2*. The *miR-2* family is widespread in invertebrates, and it is the largest family of miRNAs in the model species *Drosophila melanogaster* (8 family members), *Capitella teleta* (7 family members), *Apis mellifera* (6 family members), *Bombyx mori* (5 members) [20].

Counting redundant miRNA reads revealed that expression varies significantly among different miRNAs. In the three pre-adult stages, insect-specific miRNA (*miR-263a*) was found to be the most abundant miRNA. However, *miR-8* was the common miRNA in the unfed adult females library. Furthermore, the four libraries shared five out of the top ten most frequently occurring miRNAs: *miR-263a*, *miR-10*, *miR-184*, *bantam* and *miR-8*. In *Drosophila*, miRNA *miR-263a* confers robustness during development by protecting nascent sense organs from apoptosis [9]. Moreover, a recent study on *Nilaparvata lugens* reported that *miR-263a* was found as high abundant miRNAs in the last instar female nymph females [78]. Both *miR-8* and *miR-184* were reported in the embryos of *Drosophila* [79, 80], mosquitoes [68, 81], silk worm [82], *Schistosoma japonicum* [83], zebrafish [84], Asian seabass[85], mouse [86–88] and humans [15, 89]. This suggest a conservative developmental function for these two miRNAs across different animals. High number of reads for *miR-2941*, *miR-2943* *miR-2944a*, and *miR-2944b* in the eggs library were also observed, which indicate an embryonic roles for these insect specific miRNAs. High expression for *miR-2941* and *miR-2943* in the embryonic stage was reported previously [53, 90].

The expression of miRNAs varies across different developmental stages [82, 91]. In this study, *bantam*, *miR-8*, *miR-31* and *miR-278* were among the up-regulated miRNAs between the egg and larva stage. In *D. melanogaster*, overexpression of *bantam* induces tissue overgrowth. Related to growth, and also in *D. melanogaster*, *miR-8* and *miR-278* have been implicated in insulin receptor signaling , thus contributing to regulation of the energy balance [6]. Embryos depleted for *miR-31* can complete development, but were affected by severe segmentation defects [92]. In the gregarious phase of locust, canonical miRNAs were expressed at levels between 1.5- and 2-fold higher than in the solitary phase. The most prominent differences were found in *miR-276*, *miR-125*, and *miR-315* [34]. Interestingly, the same change in these miRNAs between the egg and the larva stage except for *miR-315* were observed. As is known, the genes that encode for miRNA are distributed across the chromosome either individually, or in clusters in which two or more miRNA genes are located within a short distance on the same segment of a chromosome [93, 94]. Therefore, it is assumed that miRNA genes located in a gene cluster are first transcribed as a single primary transcript that is subsequently processed to generate the individual miRNAs [7]. In the larvae library, very high relative expression levels of the *let-7*-complex locus (*miR-125*, *let-7* and *miR-100*), the cluster of the two mosquito-specifc miRNAs; *miR-1174* and *miR-1175*, *miR-278* and *miR-307*, *miR-305* and *miR-275*, *miR-210* and *miR-927* were observed. Down-regulation of the *miR-92* cluster (*miR-92a* and *miR-92b*) and *miR-309* cluster (*miR-309* and *miR-286*) were also observed. Similarly in *Drosophila*, *miR-309* and *miR-286* (which was processed from a single 1.5 kb primary transcript) displayed a dynamic pattern of expression in the early embryo [95].

Between the larva and the pupa, *miR-193*, *miR-277* and *miR-1890* were significantly up-regulated. *miR-34*, *miR-308*, *miR-375*, *miR-1175* and and *miR-2942* were down-regulated in the pupa after increasing in the larva stage. Again, down-regulation of the *miR-92a* cluster was noticed. The expression of the members of this cluster has been related to the embryonic development in *Ae. aegypti* [68] and *B. mori* [69].

Among the up-regulated miRNA between the pupa and the unfed adult female is *miR-1891* the mosquito-specific miRNA which displayed changes in its expression levels in the unfed adult females library, suggesting a significant role for this miRNA during development beside its function in the host response to infection [14]. In the unfed adult females library, up-regulation of *miR-34* was noticed compared to significant down-regulation in the pupae library, and a major change in *miR-989* expression for the first time. The expression of these miRNAs has been studied in different fly species, and results have shown that *miR-989* expression is restricted to females, and predominantly to the ovary in *An. anthropophagus*, *An. stephensi*, *Ae. aegypti* and *Drosophila* [52, 53, 68, 96], although it was later detected in the midgut of *An. gambiae* [7]. Both miRNAs (*miR-34* and *miR-989*) with two other miRNAs displayed changes in the expression levels during *Plasmodium* parasite invasion [7]. For unknown reasons, some miRNAs such as *miR-87* and the arthropod-specific *miR-929* showed low read counts in the four life stage libraries, suggesting that miRNAs might be involved in other process but not development. Further studies are needed to reveal the function of these miRNAs.

The predicted novel miRNAs exhibited much lower expression levels, consistent with the evidence that non-conserved miRNAs are often expressed at a lower level than conserved miRNAs [97–99].

It is important to note that the expression profile analysis in this study are based only on the sequence read counts and required further experimental validation. However, this result is a key step towards improving our understanding of the complexity and regulation mode of miRNAs in mosquitoes. Changes in the expression profiles were noted in all stages indicating a role for these small RNAs in the mosquito maturation. Knockdown or blocking the biogenesis pathway of one of these miRNAs may limit the mosquito’s development at a crucial stage thereby leading to novel approaches to combat this mosquito in the early development stages.

## Conclusion

In this study, 107 mature miRNA sequences in the four developing stages (eggs, larvae, pupae, and unfed adult females) of *An. funestus* were identified, one of the most prevalent malaria vector on the African continent using the high throughput sequencing and described their expression patterns during development.
